# Role of Immune Microenvironment in Pancreatic Ductal Adenocarcinoma: Could It Be Considered a Predictor of Prognosis?

**DOI:** 10.3390/curroncol30060417

**Published:** 2023-06-08

**Authors:** Ottavia De Simoni, Luca Dal Santo, Marco Scarpa, Giada Munari, Ylenia Camilla Spolverato, Antonio Scapinello, Sara Lonardi, Caterina Soldà, Francesca Bergamo, Alberto Fantin, Romeo Bardini, Pierluigi Pilati, Matteo Fassan, Mario Gruppo

**Affiliations:** 1 Surgical Oncology of Digestive Tract Unit, Veneto Institute of Oncology (IOV-IRCCS), 35128 Padua, Italy; pierluigi.pilati@iov.veneto.it (P.P.); mario.gruppo@iov.veneto.it (M.G.); 2 Pathology Unit, Department of Medicine, University of Padova, 35128 Padua, Italy; lucas1186dalsanto@gmail.com (L.D.S.); matteo.fassan@unipd.it (M.F.); 3 Chirurgia Generale 3, Azienda Ospedale Università Padova, 35128 Padua, Italy; marcoscarpa73@yahoo.it; 4 Veneto Institute of Oncology (IOV-IRCCS), 35128 Padua, Italy; giada.munari@iov.veneto.it; 5 General Surgery Unit, Azienda Ospedaliera di Padova, 35128 Padua, Italy; ycspolverato@gmail.com (Y.C.S.); romeo.bardini@unipd.it (R.B.); 6 Anatomy and Pathological Histology Unit, Veneto Institute of Oncology (IOV-IRCCS), 35128 Padua, Italy; antonio.scapinello@iov.veneto.it; 7 Unit of Medical Oncology 3, Veneto Institute of Oncology (IOV-IRCCS), 35128 Padua, Italy; sara.lonardi@iov.veneto.it; 8 Unit of Medical Oncology 1, Veneto Institute of Oncology (IOV-IRCCS), 35128 Padua, Italy; caterina.solda@iov.veneto.it (C.S.); francesca.bergamo@iov.veneto.it (F.B.); 9 Gastroenterology Unit, Veneto Institute of Oncology (IOV-IRCCS), 35128 Padua, Italy; alberto.fantin@iov.veneto.it

**Keywords:** pancreatic cancer, tumor microenvironment, pancreatic surgery, pancreatectomy, overall survival

## Abstract

Background: Pancreatic ductal adenocarcinoma (PDAC) is characterized by a highly immunosuppressive tumor microenvironment (TME). The aim of this study is to determine the potential significant TME immune markers of long-term survival. Methods: We retrospectively included patients with a diagnosis of resectable PDAC having undergone upfront surgery. Immunohistochemical (IHC) staining using tissue microarray for PD-L1, CD3, CD4, CD8, FOXP3, CD20, iNOS and CD163 was performed in order to characterize the TME. The primary endpoint was long-term survival, defined as the Overall Survival > 24 months from surgery. Results: A total of 38 consecutive patients were included, and 14 (36%) of them were long-term survivors. Long-term survivors showed a higher density of CD8+ lymphocytes intra- and peri-acinar (*p* = 0.08), and a higher CD8/FOXP3 intra- and peri-tumoral ratio (*p* = 0.05). A low density of intra- and peri-tumoral FOXP3 infiltration is a good predictor of long-term survival (*p* = 0.04). A significant association of the low density of intra- and peri-tumoral tumor-associated macrophages (TAMs) iNOS+ with long-term survival was detected (*p* = 0.04). Conclusions: Despite the retrospective nature and small sample size, our study showed that the high infiltration of CD8+ lymphocytes and low infiltration of FOXP3+ and TAMs iNOS+ are predictors of good prognosis. A preoperative assessment of these potential immune markers could be useful and determinant in the staging process and in PDAC management.

## 1. Introduction

Despite recent advances in the knowledge of pancreatic ductal adenocarcinoma (PDAC) carcinogenesis, PDAC remains one of the most lethal malignancies, with an overall 5-year survival rate of approximately 8% [1,2]. Nevertheless, patients who are eligible for curative surgery followed by adjuvant therapy have 5-year survival rates of approximately 20%, with a median survival time of 25 to 30 months [3,4]. Nowadays, one of the open questions in PDAC management is to define the behavior of the disease, its prognosis and its ability to respond to chemotherapy based on molecular parameters, beyond radiological staging and macroscopically visible tumor lesions. Currently, there are no standard molecular parameters capable of determining the aggressiveness of disease that could guide the decision making in PDAC treatment. In particular, there is a pressing need to decipher the underlying elements responsible for the long-term survival of patients with a diagnosis of PDAC.

Accumulated evidence has suggested that PDAC induced multiple “immune defects”, including a lack of high-quality effector cells, barriers to effector cell infiltration due to heterogeneous dense stroma, an immunosuppressive tumor microenvironment (TME) and immune checkpoint signaling [5]. It was shown in recent years that the TME plays a critical role in tumor progression and might provide the clinicians with new tools that could help to choose the most correct therapeutic pathway [6]. In fact, PDAC is considered a highly immunosuppressive and heterogeneous neoplasm, characterized by an immunosuppressive TME and a high ability to evade immune surveillance [7,8,9]. Tumor-infiltrating immune cells play an integral role in shaping the TME, and PDAC development is intertwined with multiple types of immunosuppressive cells, including regulatory T (Treg) cells, myeloid-derived suppressor cells (MDSCs) and tumor-associated macrophages (TAMs) [10,11]. In this study, we characterized the immune TME in patients with a diagnosis of PDAC, who underwent surgery with curative intent. The aim of this study was to analyze the clinical and pathological impact of the TME in patients with a diagnosis of PDAC characterized by long-term survival, defined as the Overall Survival (OS) >24 months from surgery, and to assess its potential role as a predictor of prognosis.

## 2. Materials and Methods

### 2.1. Study Design

The present study is based on a retrospective cohort including patients with resectable PDAC having undergone curative surgery at the Unit of Surgical Oncology of Digestive Tract (Veneto Institute of Oncology, IOV) between March 2018 and August 2020. Resectable patients were defined according to the NCCN Guidelines Version 2.2021 [12]. Only patients who had paraffin-embedded specimens of tumor tissue biopsies of good quality and with pathology-proven PDAC were included. Patients having undergone neoadjuvant chemotherapy were excluded. The exclusion criteria were a lack of follow up, not-curative surgery, and previous or concomitant neoplasms. Information on demographic and clinical characteristics were collected through medical records and pathological records. Every patient was followed up at the outpatient clinic every 1–3 months during the first postoperative year, and every 6 months thereafter. All included patients had a potential follow-up of at least 24 months. We considered long-term survivors patients with a diagnosis of PDAC having undergone curative surgery and a regular follow up with an OS > 24 months from surgery. The primary endpoint was a 2-year OS (long-term survival).

### 2.2. Ethical Statement

All investigations performed in relation to this manuscript were conducted according to the principles expressed in the Declaration of Helsinki. The patients signed a written consent form to have their data used for scientific purposes, and the study was approved by the local ethical committee of the Veneto Institute of Oncology (CESC IOV 2022-02).

### 2.3. Histopathological Characterization

In order to confirm tissue quality and the presence of PDAC prior to the study, the paraffin blocks available were re-evaluated by an independent pathologist (LDS) blinded to the original evaluation. The tumors were staged according to the TNM classification system for pancreatic carcinoma issued by the Union of International Cancer Control (8th edition) [13]. Intra-tumoral and peri-tumoral (e.g., detected at the invasive neoplastic front), as well as intra-epithelial lymphocytes in the tumor spots were considered; tertiary lymphoid structures (TLSs) were excluded from the evaluation.

### 2.4. Tissue Microarray (TMA) Construction

To construct the tissue microarray, the formalin-fixed, paraffin-embedded archival tissue blocks and their matching H&E-stained slides were reviewed and screened for representative tumor regions and normal pancreatic parenchyma by two gastrointestinal pathologists. The representative tumor regions included a tumor center, invasion front and areas of tumor-infiltrating lymphocytes (TILs). For each patient, four cores of the tumor and two cores of the paired normal pancreatic parenchyma were sampled from representative areas using a 1.0 mm punch. If little material was available, only three cores of the tumor and one core of normal pancreatic parenchyma were obtained. All surgical samples were processed using the Galileo CK3500 Arrayer (www.isenet.it accessed on 5 May 2023), a semiautomatic and computer-assisted tissue microarray (TMA) platform. Three different TMA blocks were produced, resulting in 237 spots for evaluation. The constructed TMA blocks were sealed with paraffin, and 3–4 mm thick slides were cut from the TMA blocks for immunohistochemical staining.

### 2.5. Immunohistochemical Staining

Immunohistochemical (IHC) staining using antibodies (clone, dilution; manufacturer) for PD-L1 (EPR1161(2), 1:50; Abcam, Cambridge, UK), CD3 (clone SP7, 1:400, Abcam, Cambridge, UK), CD4 (clone CD4/4B12, 1:100, Dako, Santa Clara, CA, USA), CD8 (clone C8/144B, 1:100, Dako, Santa Clara, CA, USA), FOXP3 (clone 236A/E7, 1:100 Abcam, Cambridge, UK), CD20 (clone L26, 1:100, Dako, Santa Clara, CA, USA ), iNOS (iNOS, 1:100, Abcam, Cambridge, UK) and CD163 (NCL-CD163CD163+; 1:100, Novocastra, Newcastle, UK) was performed in order to characterize the TME. The staining of appropriate control tissues to evaluate the specificity of all antibodies was performed.

The immunohistochemical stains for PD-L1 were evaluated for the presence of partial or complete membrane staining in tumor cells or the presence of membrane and/or cytoplasmic staining of mononuclear inflammatory cells (lymphocytes and macrophages) within the tumor and/or adjacent supporting stroma.

PD-L1 expression was measured using the CPS scoring system. The combined positive score (CPS) is calculated as the number of PD-L1-positive cells (tumor cells, lymphocytes and macrophages) divided by the total number of viable tumor cells multiplied by 100. The CPS is expressed by the following formula: CPS = (number of PD-L1–stained cells: tumor cells, lymphocytes, macrophages/total number of viable tumor cells) × 100. All samples were confirmed to include at least 100 viable tumor cells, which is regarded as adequate for PD-L1 assessment.

CD3-stained lymphocytes were counted manually in five high-power fields (HPFs) among all tumor spots and in three HPFs among all spots obtained from paired normal pancreatic parenchyma. The average lymphocyte number was calculated across HPFs. The end result was that each patient had a final score for the tumor area regarding both intra-tumoral/peri-tumoral and intra-epithelial lymphocytes. Moreover, a final score for the non-neoplastic pancreatic tissue regarding periacinar and intra-epithelial lymphocytes was obtained. The same method was applied for CD4-, CD8-, FOXP3- and CD20-stained lymphocyte evaluation.

CD163- and iNOS-stained intra-tumoral and peri-tumoral TAMs were counted manually in five HPFs among all tumor spots for each patient. Intra-epithelial, as well as intra-glandular macrophages were omitted. Finally, the average of CD163- and iNOS-stained macrophage number was calculated for each sample. Moreover, CD163- and iNOS-stained intra-acinar and periacinar macrophages were counted manually in three HPFs among all spots obtained from normal pancreatic parenchyma, obtaining the average CD163+ macrophage and iNOS+ macrophage number for each case.

### 2.6. Statistical Analysis

Statistical analyses were performed using Origin statistical program 2.6 (OriginLab, Northampton, MA, USA). Continuous data were presented as the mean (SEM) and dichotomous data were presented as the frequency (%). Independent continuous data were compared using the Mann–Whitney U test and the Kruskal–Wallis ANOVA, as appropriate. All tests were two-sided and a *p*-value less than 0.05 was considered significant. Immune marker expressions were dichotomized into high-versus-low density using an optimal threshold value, which was calculated by minimizing the Manhattan distance on the ROC curve to the left top edge of the diagram (Youden’s Index) constructed from long-term survival (OS > 24 months) data. Survival analysis was performed using the Kaplan–Meier method, and the curves were compared with a log-rank test [14].

## 3. Results

### 3.1. Patient Characteristics

A total of 38 patients with a diagnosis of PDAC having undergone curative surgery followed by adjuvant chemotherapy, were retrospectively included in the analysis. The median age at diagnosis was 72 years (range 52–83). The median BMI was 23.2 kg/m^2^ (range 15.2–28.6). Demographic and clinical data are summarized in Table 1.

Pancreaticoduodenectomy was performed in 31 (81%) patients and distal pancreatectomy in 7 (19%) patients. Radicality (R0) was reached in 30 (79%) patients. The pathological data are summarized in Table 2.

The median overall survival (OS) was 17 months (range 9–53). A 1-year OS made up 60%, and 2-year OS 36%. After a median follow-up of 16.5 months (range 3–53), 25 (65%) patients had local or distant recurrence and 24 (63%) died from the disease. According to the above-mentioned definition, we identified among these patients 14 (36%) long-term survivors. As shown in Table 1, no differences in terms of age, gender, BMI and the Karnofsky Score were observed between long-term survivors and no-long-term survivors. Moreover, no differences in terms of pathological data were reported between the two groups of patients. The median overall survival (OS) in long-term survivor patients was 34 months (range 27–53).

### 3.2. Immune Tumor Microenvironment in Long-Term Survivors

The number of all the subclasses of lymphocytes was significantly higher in the tumor and peri-tumoral compartment, compared to the intra-epithelial compartment and normal pancreatic parenchyma (CD3+ lymphocytes 97.7 vs. 2.66 vs. 31.68, *p* < 0.001; CD4+ lymphocytes 44.7 vs. 0.51 vs. 11, *p* < 0.001; CD8+ lymphocytes 62.74 vs. 2.32 vs. 26.3, *p* < 0.001; CD20+ lymphocytes 44.37 vs. 0.04 vs. 5.49, *p* < 0.001, respectively).

Among lymphocytes, the number of CD8+ lymphocytes was higher compared to that of CD4+ lymphocytes, both in the tumor and peri-tumoral compartments and normal pancreatic parenchyma (49.6 vs. 37.8, *p* = 0.04, 26.3 vs. 11, *p* < 0.001, respectively). FOXP3+ expression was frequently observed in tumor and peri-tumoral compartments compared to normal pancreatic parenchyma (13.8 vs. 2, *p* < 0.001).

**Figure 1:**
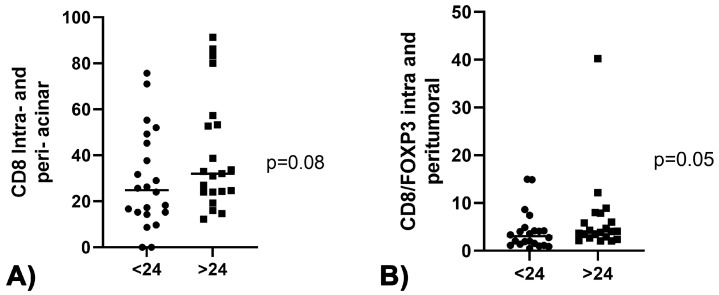
(**A**) Association between CD8 intra- and peri-acinar lymphocytes and OS > 24 months. (**B**) Association between CD8/FOXP3 intra- and peri-tumoral ratio and OS > 24 months.

CD3+, CD4+ and CD20+ lymphocyte infiltration did not show any significant association with PDAC patients’ long-term survival. Long-term survivors showed a higher density of CD8 intra- and peri-acinar lymphocytes (*p* = 0.08), as shown in Figure 1A. Moreover, the ratio CD8/FOXP3, intra- and peri-tumoral, was higher in patients with OS > 24 months (*p* = 0.05), as reported in Figure 1B.

**Figure 2:**
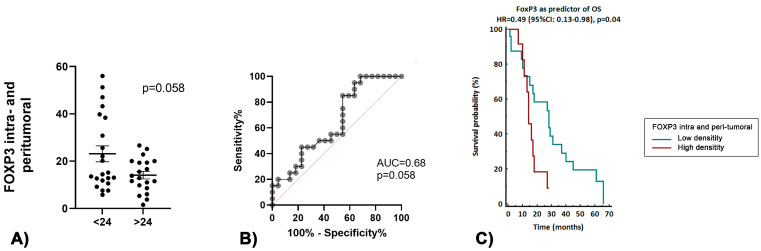
(**A**) Association between TILs FOXP3+ and OS > 24 months. (**B**) Accuracy of TILs FOXP3+ as a predictor of OS > 24 months. (**C**) Survival analyses based on the dichotomized density of the TIL FOXP3+.

PDL-1 expression did not show any significant association with PDAC patients’ long-term survival (21.1% in no long-term survivors vs. 10.5% in long-term survivors, *p* = 0.8). In long-term survivors, low intra- and peri-tumoral FOXP3+ cell infiltration was associated with a better OS (*p* = 0.05, Figure 2A). The ROC curve analysis revealed that a lower infiltration of FOXP3+ had a good accuracy in predicting long-term survival (AUC 0.68, *p* = 0.058), as shown in Figure 2B. Survival analysis showed an association of low density of intra- and peri-tumoral FOXP3+ with a significantly increased OS (*p* = 0.04, Figure 2C).

**Figure 3:**
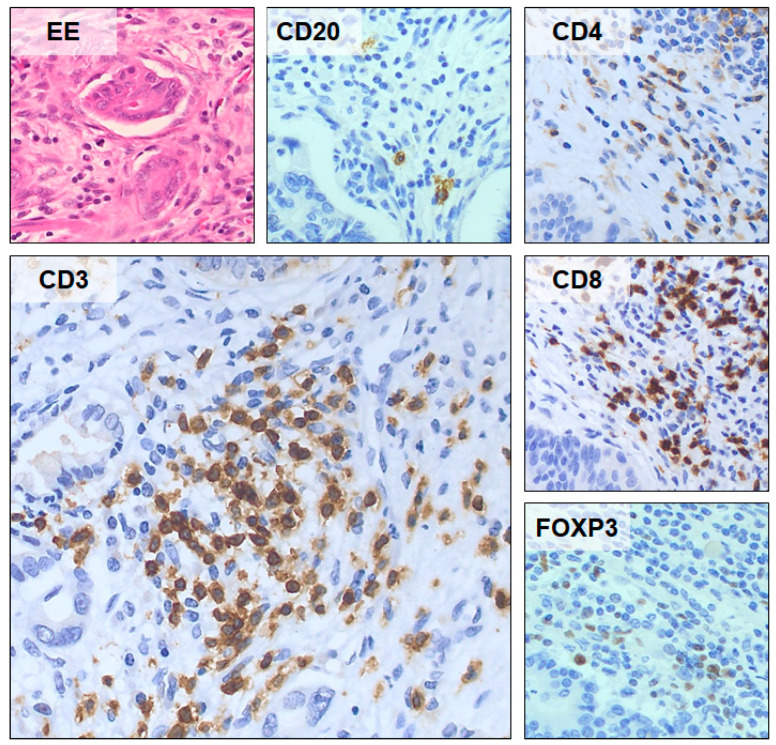
Representative example of the density expression of the TILs CD20+, CD4+, CD3+, CD8+ and FOXP3+ in tumoral pancreatic parenchyma. Images taken at 40× magnification.

Figure 3 shows a representative example of the density expression of the TILsCD20+, CD4+, CD3+, CD8+ and FOXP3+ in tumoral pancreatic parenchyma.

**Figure 4:**
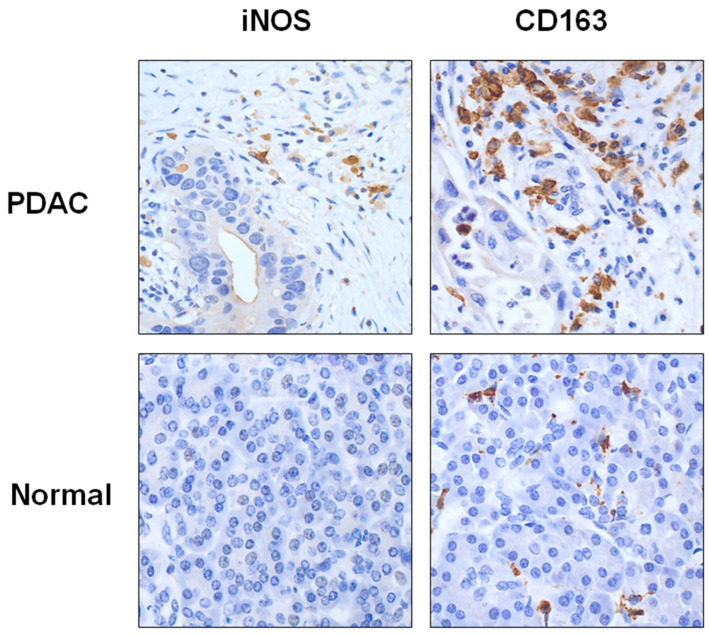
Representative example of the density expression of TAMs INOS and CD163+ in tumoral and normal pancreatic parenchyma. Images taken at 40× magnification.

The number of both iNOS+ and CD163+ macrophages was significantly higher in the tumor and peri-tumoral compartment, compared to normal pancreatic parenchyma (iNOS+ macrophages count was 5 vs. 2.5, *p* = 0.009; CD163+ macrophages count was 92 vs. 66; *p* = 0.002; respectively), as shown in Figure 4.

Among macrophages, the number of CD163+ macrophages was higher compared to that of iNOS+ macrophages, both in the tumor and peri-tumoral compartments and normal pancreatic parenchyma (92 vs. 5, *p* < 0.001; 66 vs. 2.5, *p* < 0.001, respectively), as shown in Figure 4.

**Figure 5:**
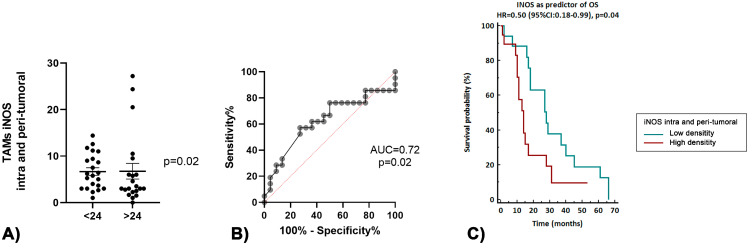
(**A**) Association between the TAM INOS, intra- and peri-tumoral, and OS > 24 months. (**B**) Accuracy of the TAM INOS, intra- and peri-tumoral, as a predictor of OS > 24 months. (**C**) Survival analyses based on the dichotomized density of the TAM INOS, intra- and peri-tumoral.

Long-term survivors showed a lower density of TAMs iNOS+ (*p* = 0.05), as shown in Figure 5A, and the ROC curve analysis demonstrated that a lower infiltration of TAMs iNOS+ was a good predictor of increased survival (AUC 0.69, *p* = 0.05, Figure 5B). Survival analysis demonstrated an significant association between the low density of intra- and peri-tumoral TAMs iNOS+ and increased OS (*p* = 0.04, Figure 5C).

**Figure 6:**
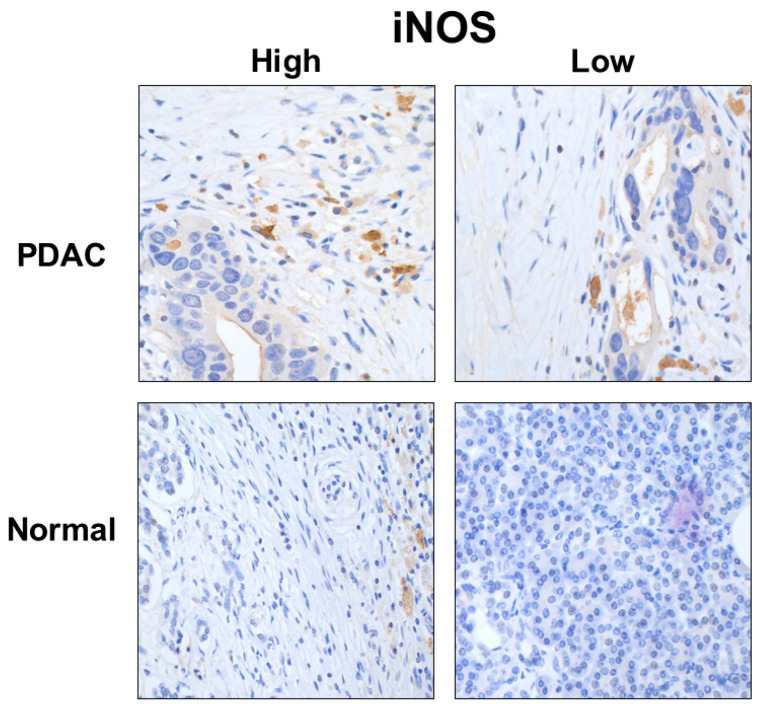
Representative example of the high- and low-density expression of the TAM INOS, intra- and peri-tumoral, in tumoral and normal pancreatic parenchyma. Images taken at 40× magnification.

Figure 6 shows a representative example of the high- and low-density expression of the TAM iNOS in PDAC.

## 4. Discussion

PDAC is considered one of the most aggressive neoplasms and its prognosis remains poor [1,2]. The responsibility for the poor prognosis is largely attributable to a highly immunosuppressive and heterogeneous behavior of the neoplasm, characterized by an immunosuppressive TME and a high ability to evade immune surveillance [7,8,9]. Moreover, the PDAC TME was postulated to limit immune cell infiltration and impair its function within the tumor [15,16].

The composition of the TME is complex and often shows different characteristics, according to the tissue from which the tumor arises, the carcinogenetic pathways involved and tumor stage.

PDAC development is intertwined with multiple types of immunosuppressive cells, including regulatory T (Treg) cells, myeloid-derived suppressor cells (MDSCs) and tumor-associated macrophages (TAMs), and leads to an inherently immunosuppressed TME [17]. Moreover, a unique feature of PDAC is the presence of a desmoplastic stroma that accounts for the majority of the tumor volume. The stromal compartment, also referred to as the TME, consists of cancer-associated fibroblasts (CAFs) and immune cells that are embedded in an extracellular matrix rich in cytokines and soluble growth factors. The role of the stromal compartment in PDAC progression is complex, with studies supporting both tumor-promoting and tumor-restrictive roles [18,19,20,21].

Finally, the role of inflammatory cells in human cancer is controversial. Although the presence of infiltrating inflammatory cells in a tumor mass represents the basis of the “immunosurveillance” against tumor growth, many recent studies indicate that inflammatory components of a developing neoplasm can provide a useful means for cancer growth and spread, mostly by potentiating extracellular matrix remodeling and angiogenesis [22]. PDAC stroma is mainly composed of extracellular matrix, myofibroblasts and various immune cells, with a predominance of TILs and TAMs [23]. Many authors have shown that leukocytes (T cells, NK cells, B cells, macrophages and granulocytes, including eosinophils, mast cells and neutrophils) are a prominent component of PDAC tumors and contribute to the formation of a TME that is ultimately permissive of tumor progression [24]. The main mechanism of immune evasion of PDAC was likely the lack of potent immunogenic tumor antigens (antigenicity), due to poor antigen presentation [25]. The degree of T cell infiltration, its high immunologic heterogenicity and specific variability in distribution within the pancreatic parenchyma, plays a key role in this action [26,27,28]. Considering that CD4+ and CD8+ T cells enclose several subpopulations with specific regulation, effector cytokine production, and functions in immunity, the differential and complexes roles of T cells in PDAC are dependent on the spatial distribution, type of subpopulation involved, and accompanying macrophage infiltration [29,30]. On the other hand, TAMs mediate immunosuppression, angiogenesis and promote tumor progression by releasing cytokines, proteases and growth factors [31,32]. Moreover, TAMs have been shown to drive resistance to gemcitabine-based chemotherapy and targeted antiangiogenic treatment, and to impair the efficacy of therapeutic irradiation [33,34].

Our data did not show any association between CD3+ and CD4+ lymphocytes and long survival. Instead, there is an association between a high infiltrate density of the TIL CD8 and long survival. It is known in literature that the TIL CD8+ infiltration of many types of cancer tissues can be correlated with long-term survival, indicating an active immunological response against malignant cells [35,36,37].

Fukunaga et al. demonstrated that TILs CD4+ and CD8+ alone cannot be considered a significant prognostic indicator in PDAC [38]. However, the overall survival rate of the CD8 (+) group was reported by many authors, higher than that of the CD8 (−) group, probably due to the activity of these T cells that attack tumor cells as foreign bodies [29,38,39]. In addition to the combination of CD4Thigh and CD8Thigh, a lower tumor-infiltrating %Treg was reported as a close association with longer survival [29]. Lundgren et al. underlined that the strongest prognostic impact associated with the distances from each cancer cell and the nearest lymphocyte was seen for TILs CD4+ and CD8+ [39]. Our data confirmed this observation, as we found that the strongest prognostic impact was found in TILs, peri-tumoral and intra-epithelial. A similar association was found by Masugi et al., who underlined the prognostic importance of the topographic infiltration patterns of CD8+ cell infiltration in the tumor center [40].

Our data showed that FOXP3+ expression was frequently observed in tumor and peri-tumoral compartments, suggesting that regulatory CD4+ lymphocytes had a greater tendency to infiltrate tumoral parenchyma. Furthermore, in our series, low intra- and peri-tumoral FOXP3+ cell infiltration, a transcription factor critical for Treg development and function, was associated with a trend of the significance of long-term survival.

The low infiltration of Treg TIL cell subsets, with potential immunosuppressive capacities, was significantly associated with both a longer OS and disease-free survival [41,42,43]. Treg TILs are well-known inhibitors of antitumor immunity and can downgrade the activity of TILs CD4+, TILs CD8+ and NK cells [44]. The mechanisms suggested for the TIL Treg-cell-mediated immune suppression include the direct elimination of, or competition with, effector T cells for access to antigen-presenting cells [44]. Other authors, such as Wang et al., found that FOXP3 was significantly represented in poorly differentiated tumors [45]. As underlined by Ino et al., the infiltration of PDAC by TILs CD4þT or CD8Þt alone was not sufficiently associated with a longer survival; however, tumor-infiltrating TILs CD4þT-high/CD8þT-high/%Treg-low were an independent prognostic survival factor [29]. Furthermore, De Monte et al. found that Th2 rather than Th1 cells predominantly infiltrate PDAC, and that the Th2/Th1 tumor-infiltrating ratio was independently predictive of survival [46]. Therefore, our data and those from literature suggest that a tumor microenvironment with a low Treg TIL infiltration is a favorable marker of survival. Nevertheless, the role of Tregs lymphocytes, commonly suggested to be immune inhibitory, remained controversial in PDAC. In fact, some authors, reported that FOXP3 expression in pancreatic cancer tumors can be induced by activation per se, without being linked to an immunosuppressive function [39,47,48].

In our series, the low infiltration of TAMs iNOS+ is a significant predictor of long-term survival. TAMs are the most abundant tumor-infiltrating immune cells in PDAC [49]. In the context of cancer immunity, two important subsets of macrophages have been recognized: M1 (iNOS+) and M2 (CD163+) macrophages. M1 macrophages play a principal role as immune-stimulatory macrophages, whereas M2 macrophages are immune-regulatory and sustain or promote cancer growth by inhibiting anticancer immunity [50]. However, within the pancreas, there are tissue resident macrophages (TRMs) that reside in the pancreatic islets, known to be the expression of an inflammatory nature that should be considered within the M1 category [51]. TAMs receive signals from different cells within the tumor microenvironment and release various growth factors and cytokines, promoting tumor cell invasion, inducing angiogenesis, suppressing antitumor immunity and facilitating lymphatic vessel invasion and tumor cell metastasis [52,53,54,55,56]. In most cases and our results, theM2 TAMs were predominant among tumor-infiltrating macrophages, suggesting a contribution to sustained cancer growth. On the contrary, the M1 TAMs were predominant in non-cancerous inflammatory region surrounding the area of cancer, releasing gamma interferon and other inflammatory cytokines [29,57]. Several studies have reported that a high amount of tumor-infiltrating macrophages is correlated with a poor outcome in various cancers [58,59]. The number of infiltrating macrophages expressing CD163 or CD204 had a stronger correlation with advanced T category and lymph node metastasis and promote a Th2, pro-tumorigenic or immunosuppressive response [60,61,62,63]. Our findings supply the hypothesis that M1 TAMs might play a role in patient prognosis, promoting carcinogenesis and neoplastic progression, and a low expression in peritumoral tissue that could positively affect the OS.

Despite the evident limits of this study, such as the retrospective nature and small sample size, our study demonstrates a significant association between some TME immune markers and long-term survival. In particular, a low infiltration of intra- and peri-tumoral FOXP3+ TILs and intra- and peri-tumoral TAM iNOS were associated with prolonged survival. More perspective studies and larger series are needed to validate our findings and, possibly, confirm the feasibility of the immunological analysis on the preoperative biopsy to allow for the preoperative examination of these immune-related signatures [64]. As underlined by Wang et al., EUS-FNB may provide sufficient tissue to allow for the preoperative examination of the immune-related signature, finding that the expression of these immune cells was significantly correlated with the expression determined in the resection specimens [65]. Not least, accumulating evidence illustrates the importance of achieving a tailored therapeutic approach, based upon the comprehension of the multi-faceted roles of the complex tumor microenvironment components and mutational status in PDAC that currently only a preoperative histological diagnosis can offer [17]. Likewise, the immune microenvironment that can be studied through preoperative biopsy may assume a key role in describing disease behavior in the future, also affecting therapeutic decisions. The therapeutic investment of a patient diagnosed with PDAC should be conditioned by disease behavior, which varies greatly from case to case and is certainly conditioned by the immune TME.

## 5. Conclusions

TME immune markers, as other potential molecular markers, could represent valid tools in predicting tumor aggressiveness and prognosis, with the ability to improve the staging process and selection of the proper therapeutic pathway in patients with a diagnosis of PDAC.

## Figures and Tables

**Table 1:** Demographic preoperative clinical data.

	Total Patients	No Long-Term Survivor	Long-Term Survivor	*p*-Value
**Number of patients** (*n*)	38	24	14	
**Age,** median (range)	72 (52–83)	72.16 (53–83)	70 (53–83)	0.18
**Sex,** *n* (%)				0.9
Male	23 (60)	14 (58)	9 (64)	
Female	15 (40)	10 (41.6)	5 (35)	
**BMI,** median (range)	23.2 (15.2–28.6)	24.75 (20–28)	20 (15.2–28.6)	0.16
**Karnofsky Score,** median (range)	98.10 (80–100)	97.10 (80–100)	98.20 (80–100)	0.9
**Diabetes**, *n* (%)	12 (31)	7 (29)	5 (35)	0.9
***Preoperative Ca 19-9***, *median (Ku/L)*	4851 (2–39,089)	3885 (0–39,089)	6779 (56–37,800)	0.69

**Table 2:** Pathological data.

	Total Patients	No Long-Term Survivor (*n* = 24)	Long-Term Survivor (*n* = 14)	*p*-Value
**Tumor location,** *n* (%)				0.22
Head	31 (81)	18 (75)	13 (92)	
Body–tail	7 (19)	6 (25)	1 (8)	
**Type of surgery,** *n* (%)				0.22
Pancreatoduodenectomy	31 (81)	18 (75)	13 (92)	
Distal pancreatectomy	7 (19)	6 (25)	1 (8)	
**Radicality,** *n* (%)				0.99
R0	30 (79)	19 (79)	11 (78)	
R1	8 (21)	5 (21)	3 (21)	
**Tumor differentiation,** *n* (%)				0.51
G1–2	12 (31)	7 (30)	5 (35)	
G3	26 (69)	17 (70)	9 (65)	
***pT category*,** *n* (%)				0.15
*T1*	2 (5)	2 (8)	0	
*T2*	13 (34)	11 (45)	2 (14)	
*T3*	23 (60)	11 (45)	12 (86)	
**Lymph-nodal status,** *n* (%)				0.14
N0	7 (19)	3 (12)	4 (28)	
N+	31 (81)	21 (88)	10 (72)	
**Lymph node retrieved,** median (range)	19.07 (4–51)	18.26 (4–51)	18.9 (6–30)	0.65
**Microvascular invasion**, *n* (%)	35 (92)	21 (87)	14 (100)	0.26
**Perineural invasion**, *n* (%)	38 (100)	24 (100)	14 (100)	0.99

## Data Availability

The data presented in this study is available in this article.
